# Relationship between Parenting Educational Styles and Well-Being in Families with Autistic Children: A Systematic Review

**DOI:** 10.3390/ejihpe14060101

**Published:** 2024-05-30

**Authors:** Elena Benseny Delgado, Wenceslao Peñate Castro, Alicia Díaz Megolla

**Affiliations:** 1 Departament of Psychology, Sociology and Social Work, Universidad de Las Palmas de Gran Canaria, Campus de Tafira, 35001 Las Palmas de Gran Canaria, Spain; 2 Faculty of Psychology and Speech Therapy, Universidad de La Laguna, Campus de Guajara, 38200 La Laguna, Spain; 3 Department of Clinical Psychology, Psychobiology and Methodology, Universidad de La Laguna, Campus de Guajara, 38200 La Laguna, Spain; wpenate@ull.edu.es; 4 Department of Education, Universidad de Las Palmas de Gran Canaria, Campus del Obelisco, 35001 Las Palmas de Gran Canaria, Spain; alicia.diaz@ulpgc.es

**Keywords:** autism spectrum disorder, parenting styles, family well-being, quality of life, satisfaction with the parental role

## Abstract

The prevalence of diagnosed cases of autism has increased rapidly, which has raised interest in studying the variables related to the well-being of these families. The purpose of this paper is to review the recent literature on other variables related to family well-being, such as parenting styles. We conducted a systematic review using the PRISMA check list and bias assessment with the aim of analyzing if the concepts of autism, well-being and parenting style are related. We screened 755 references from relevant databases like Scopus, Pubmed, PscyInfo EBSCO, Web of Science and Dialnet, updated on May 2024. Sixteen full text articles and abstracts were read. It was identified that the authoritative parenting style, as well as those based on warmth, establishing relationships and emotional bonding, and low expressed emotion were positively related to family well-being. On the other hand, authoritarian, permissive and overprotective styles, as well as critical, punishing and training-based, were negatively associated with well-being and quality of family life.

## 1. Introduction

The growing increase in the prevalence of Autism Spectrum Disorders (ASDs) in recent years has become a topic of research including primary research, meta-analyses, and systematic reviews [1]. Among the highlights of these studies is the presence of high levels of stress in parents [2,3,4,5] and the need to identify the variables that contribute to family well-being as facilitators of the proper development of these children [6]. Authoritarian parenting styles mediated the relationship between parenting relationship satisfaction and child externalizing behaviors [7]. Some parenting practices are associated with lower or higher levels of externalizing behaviors in autistic children. Internalizing behaviors include symptoms associated with anxiety, mood conditions, and somatization, and externalizing behaviors include behaviors that others may find challenging like aggression, non-compliance, tantrums [8]. Stress-resilient attributes of parents with children with developmental disorders are considered one of the factors that have a decisive influence on the behavior of parents related to raising their children and that affect greater well-being and life satisfaction [9]. Parents raising a child with ASD face daily challenges in meeting their specific needs [10]. The unique demands of autism require parents to reorganize their parenting responsibilities and roles [11], which can affect family life [12]. Families are a key factor in inclusive education because their children can play an important role in the inclusion of other children with ASD [13]. Likewise, the sense of parental competence in these cases is related to their expectations and family support needs [14].

There is little research that studies family well-being in the ASD population. Satisfaction with parental roles and authoritative styles is related to the management of stressors, which may benefit the harmonious development of children with ASD in a healthy family environment [15,16,17]. The temperament of children can be conditioned by maturation and environmental aspects [18], hence the importance of contemplating a transactional model that emphasizes the interaction of the child with his/her environment, in a relationship of mutual influence [19]. Although most of the research on parenting has assumed that parenting style influences children’s adjustment, it has also been suggested that the relationship may be bidirectional such that children’s adjustment can influence a parent’s child-rearing practices [20]. Moreover, some tendencies direct their attention towards prenatal pedagogy, in view of the possibility that the mother, with her thoughts, feelings, and way of life, is educating the child before birth [21]. On this matter, some authors highlight the need for interdisciplinary research to address ASD-related childbearing [22]. Furthermore, executive functioning in children with ASD is linked with the use of adaptative emotion regulation strategies [23]. Such evidence makes research into parental satisfaction and parenting styles more than reasonable.

Although we cannot establish a causal relationship between the symptomatology of autism and parenting styles, as it is a complex disorder with a neurobiological basis [24], the influence of the lack of reciprocal relationships and communication of children with ASD on the parenting styles of their parents has been studied. A decrease in warmth and more protective and controlling behaviors towards their children were observed [19,25]. In addition, positive parenting and the quality of parent–child interaction have been linked to higher social competence in children with ASD [26]. On the other hand, parenting style can affect parents’ stress levels, as well as their ability to cope with their children’s behaviors [20]. As a consequence, it would be interesting to analyze these variables.

Taking into account the above, our objective is to identify, through a systematic review, the relationship between parenting style and the well-being of families with children with ASD. In this review, we will analyze the concepts of parenting styles, educational styles, and/or parenting styles interchangeably. We consider the diagnostic change of the last 10 years produced by the publication of Diagnostic and Statistical Manual of Mental Disorders (DSM 5) [27].

## 2. Materials and Methods

This systematic review is based on the PRISMA checklist [28]. First, the identification, selection, eligibility, inclusion, and evaluation of biases were carried out to obtain relevant articles, and then, the references were analyzed using the Mendeley (Desktop Version 1.19.8 @ 2008-2020) bibliographic manager.

### 2.1. Participants

Participants were parents, primary caregivers of children with and without ASD. The age of the children ranged from 2 to 22 years. The samples belong to different countries: USA, India, Indonesia, Thailand, Japan, Italy, Portugal, Greece, Belgium, Netherlands, China, Hong Kong, and United Kingdom. The age of parents was described in averages in 11 of the studies, older than 18 years in the study by Likhitweerawong et al. [29], and Dieleman et al. [30] is the only study where both the age of the parents and the diagnosis of the children are specified. Parental age is not specified in the studies by Walsh et al. [31], Charman et al. [32], and Tripathi [33]. Other studies included siblings without a diagnosis.

### 2.2. Diagnosis

Of the 16 studies, 5 were based on the DSM 5 [27] diagnostic criteria for ASD and another 3 on the ADOS assessment instrument, using the DSM-IV [27] diagnostic criteria as a reference. In all cases, the diagnosis was made by a mental health professional.

### 2.3. Selection Criteria

#### 2.3.1. Inclusion

- Studies that relate parenting style to the well-being, satisfaction, and/or quality of life of families with children with ASD.
- Published in the last 10 years including the diagnostic change from DSM IV to DSM 5 [27].
- Written in Spanish or English.
- Empirical articles (qualitative and/or quantitative).

#### 2.3.2. Exclusion

- Articles that referred to disability but did not specify ASD.
- Those that did not include all three variables (family well-being, parenting style, and ASD).
- Those that considered child satisfaction or marital satisfaction; those that included only satisfaction with social or medical services were eliminated.

### 2.4. Identification and Selection of Studies

The search was conducted on the following search engines: Scopus (248 articles), Pubmed (69), PsycInfo (EBSCO) (22), Web of Science (WOS) Core Collection (305), and Dialnet (109). Different keywords were introduced as “Autism” can include terms such as “ASD” (Autism Syndrome Disorder), “Asperger’s”, “Autistic Disorder”; Parenting styles or parenting styles “teaching style” and satisfaction with the parental role. We used the Mendeley—Reference Management Software. Searches were conducted on March 2023 and updated on May 2024. Finally, a final search was performed manually. For the application of the selection criteria, the first author performed the initial screening. In case of doubt, the other authors were consulted, and the decision was made by consensus.

### 2.5. Bias Assessment

The first author carried out the initial screening, and the second authors carried out the criteria of safety, inclusion, exclusion, and elimination of the doubts that arose. The methodological evaluation was carried out by applying the STROBE bias analysis scale [34]. In the case of qualitative studies, the items were analyzed when applicable.

## 3. Results

**Figure 1:**
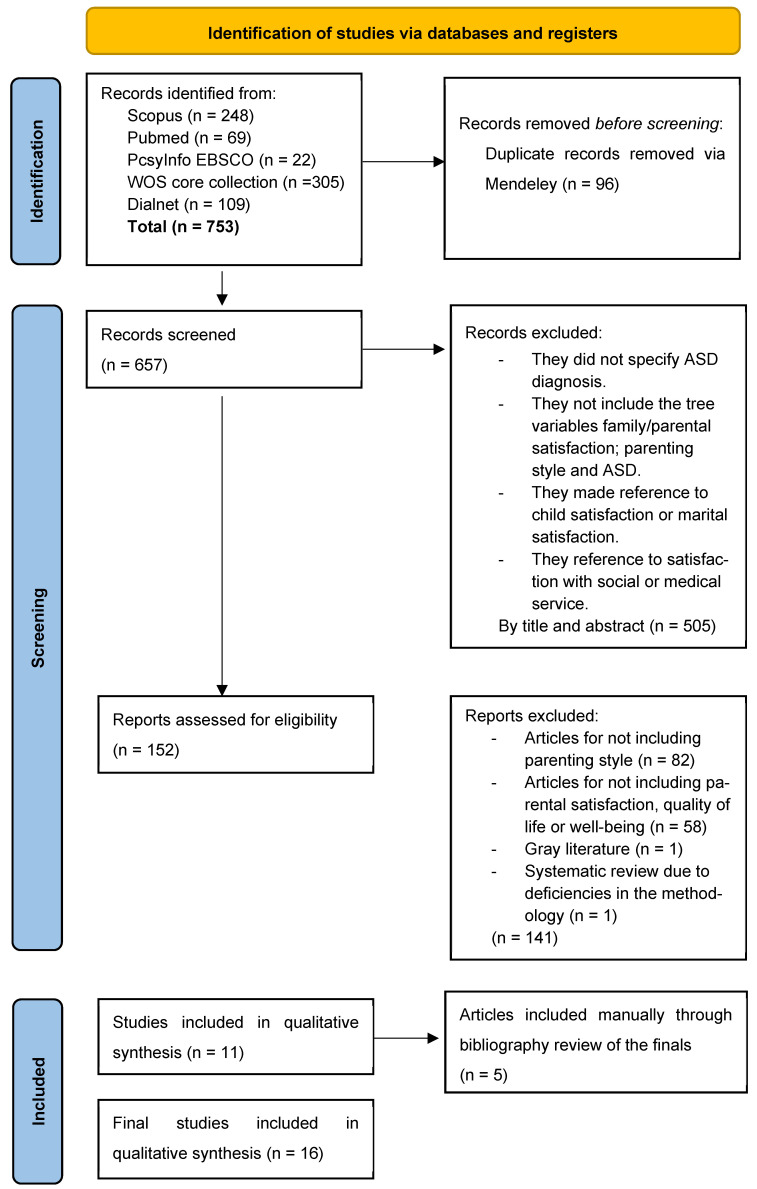
PRISMA 2020 flow diagram.

A total of 657 references were initially identified after removing duplicates. Of these, 16 were finally selected. Flow chart and selection process are described in Figure 1.

### 3.1. Eligibility

The references were classified into three groups: those that included the concept of parental satisfaction, parental well-being, or quality of life along with autism; another group with parenting styles, parenting styles, and autism; and a third with the three key concepts: autism, parenting styles, and satisfaction with the parental role.

Subsequently, those that included only ASD and parental satisfaction and those that only related ASD and parenting styles were eliminated, selecting only those that included all three concepts.

### 3.2. Methodological Strengths and Weaknesses

**Figure 2:**
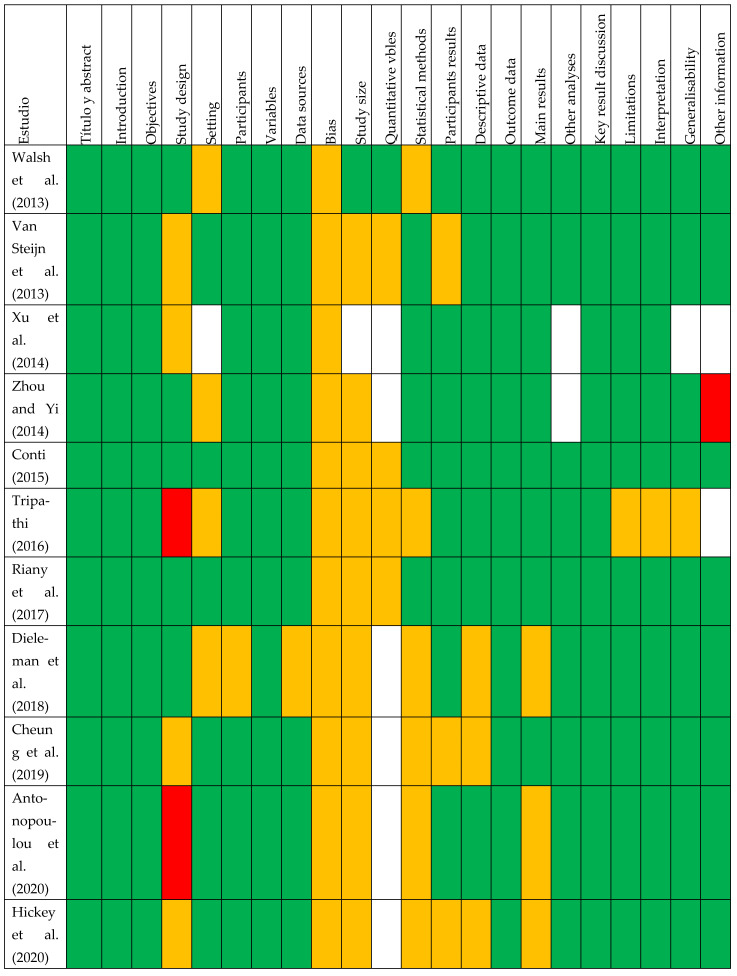

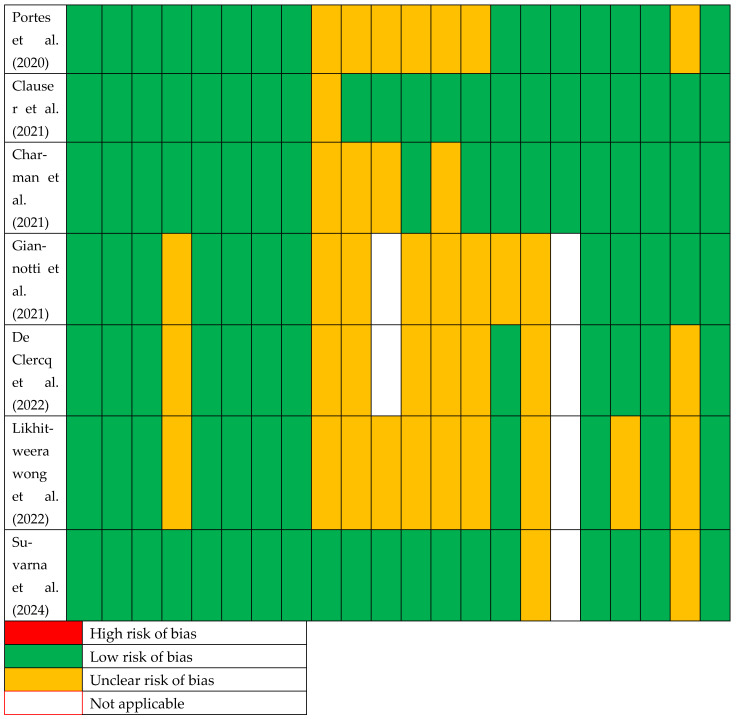
STROBE bias analysis sorted by year of publication [6,8,17,20,29,30,31,32,33,35,36,37,38,39,41,42,43,44].

**Figure 3:**
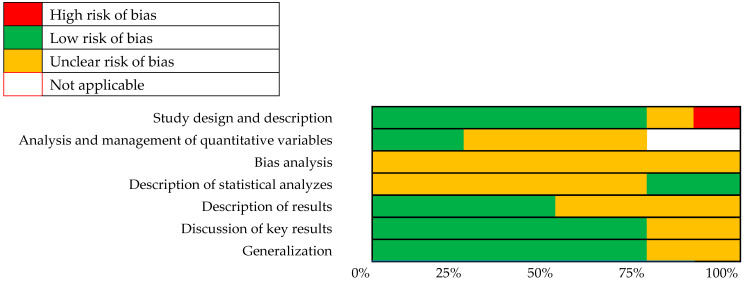
Summary risk of bias graph of included studies according to STROBE scale components.

To determine the methodological quality, the studies were analyzed using the STROBE scale [34] (Figure 2). Most studies met the STROBE criteria, highlighting participant descriptions, variables, data sources, outcome data, key outcomes, limitations, and interpretation of results. The greatest weaknesses at the methodological level were focused on the analysis of biases, the management of quantitative variables, and the description of the statistical analysis and the results. The summary graph of risks of bias of the included studies according to the components of the STROBE scale shows high-risk of bias studies in red, low-risk studies in green, yellow shows unclear risk of bias and white does not applicable (Figure 3).

Making a specific analysis and taking into account the limitations pointed out in the studies, the main methodological problems are based on not being able to establish causal relationships because they are descriptive and associative observational studies. Thus, the works of Antonopoulou [35], Cheung [36], Dieleman [30], Riany [37], and Zhou and Yi [17] are qualitative case studies with limitations to establish causal relationships. The study by Clauser et al. [20] could be biased as it was based on parents’ perceptions, which could pose a threat to internal validity. In the study by Van Steijn et al. [38], the objective speaks of influence, and its design (correlation analysis and post hoc analysis) does not allow it. Another example of problems when it comes to establishing the effect can be found in de Clercq et al. [39], as it is not clear whether parental stress drives Expressed Emotion (EE) or whether EE is a determinant of stress. Suvarna et al. [8] include in their limitations that a choice was made a priori to focus on only quantitative articles and qualitative studies and synthesis such as meta-synthesis may be valuable in the future to provide an in-depth understanding of particular behaviors [40].

Studies that have shown shortcomings in defining participants or failing to explain the loss of participants are those by Charman et al. [32], Cheung et al. [36], De Clercq et al. [39], Dieleman et al. [30], Giannotti et al. [41], Hickey et al. [6], Likhitweerawong et al. [29], Portes et al. [42], Van Steijn et al. [38]. On the other hand, the method of data collection in Clauser et al. [20] could also be considered a limitation with a relatively small sample size, just as they point out.

None of the studies explicitly identify a source of bias identification. Antonopoulou et al. [35] and Tripathi [33], do not explicitly report on design of their studies. The studies that have the most shortcomings in showing descriptive data in the results are those of Cheung et al. [36], De Clercq et al. [39], Dieleman et al. [30], Giannotti et al. [41], Hickey et al. [6], Likhitweerawong et al. [29], and Portes et al. [42]. Studies that do not dispute the generalizability of their results are those by De Clercq et al. [39], Likhitweerawong et al. [29], Portes et al. [42], Tripathi [33], and Clauser et al. [20].

Taking into account the above, it seems that the studies with the most methodological strengths are those of Walsh et al. [31] and Conti [43], and those with the most weaknesses are those of Dieleman et al. [30], Likhitweerawong et al. [29], Tripathi [33], and Xu et al. [44], this article having the most biases such as the description of the setting, the explanation of how the size of the study was reached and the quantitative variables, the description of the main results, and generalizability (see Figure 2).

### 3.3. Summary of Extracted Data

Table 1 shows the summary of the articles reviewed, classified by citation, participants, objectives, and results. Results related to the objective of this review are noted. 

### 3.4. Relationships between Study Variables

Responding to our objective (relationship between well-being and parenting style in ASD), we can determine that family well-being is positively related to the authoritative style, warmth, relationship building, affective bonding, priority in relationship building, and low EE. Higher levels of authoritarian parenting and parenting behaviors, including harsh and psychological control, are associated with higher levels of externalizing behaviors, whereas parenting behaviors including warmth and behavioral control are associated with lower levels of externalizing behaviors (Suvarna et al. [8]). In the same way, permissive parenting style is associated with higher levels of externalizing behaviors. Likewise, styles based on overprotection, permissiveness, criticism, authoritarianism, punishing, training-based, and high EE are related to poorer family satisfaction and well-being, frustration, stress, and anxiety in parents that are reflected in children and aggravate the symptoms of ASD. This relationship is present in all studies except that of Xu et al. [44], in which the authoritative style correlated with more behavioral problems and parental depression than the authoritarian and permissive style.

Family well-being was negatively associated with anxiety and stress, with parents of children with ASD experiencing significantly higher levels of stress (Antonopoulou et al. [35] and Likhitweerawong et al. [29]), less personal freedom, and more social isolation (De Clercq et al. [39] and Dieleman et al. [30]), compared to parents of children without ASD (Riany et al. [37]). When the mother’s level of parenting stress is high, this stress can spill over into the couple’s relationship and result in fathers being less involved and less warm with their children, while when it is low, the mother facilitates the father’s warmth by planning family activities (Hickey et al. [6]).

Stress was related to parenting styles; the more protective parents were, the more stress they experienced, and vice versa (Walsh et al. [31]). In addition, more permissive and less authoritative styles were associated with higher levels of stress (Tripathi [33]). Similarly, an authoritative and conscious parenting style was related to lower levels of parental stress, less perceived discrimination for having a child with ASD (affiliation stigma), and fewer behavioral problems of their children (Cheung et al. [36]).

Parents of autistic children tended to express more criticism and less warmth towards their children, which was associated with frustration and stress in key psychological areas, such as autonomy, relationships, and competence (De Clercq et al. [39]). Parents who prioritized training showed more stress and frustration if they did not observe remission of ASD symptoms in their children (Zhou and Yi. [17]). Family stress levels were associated with high EE, which, in turn, was related to insensitive parenting behaviors that cause challenging behaviors in their children (De Clercq et al. [39]).

Lower levels of stress were associated with more sensitive, responsive, and affectionate parenting by both mothers and fathers (Cheung et al. [36]). A negative association between anxiety and authoritative parenting style was observed in parents of children with high-functioning autism (Antonopoulou et al. [35]).

On the other hand, parents’ parenting styles seemed to be influenced by factors related to their children’s behavior and the stress that this behavior can generate (Walsh et al. [31] and Tripathi [33]). These factors included overprotective parenting styles and manifestations of grief on the part of children.

It is suggested that the child’s pathology may lead parents to adopt a permissive parenting style rather than other styles (Van Steijn et al. [38]). This style may be related to cognitive and social development problems in children with ASD and ADHD (Van Steijn et al. [38]). The severity of the disorder and adolescence were associated with permissive and authoritarian parenting styles (Tripathi [33]). On the other hand, Clauser et al. [20] revealed that ASD severity and parenting style contributed to externalizing behaviors of their children but did not have a significant impact on internalizing behaviors. Overall, we highlight the complex interplay between children with ASD, parenting styles, and the severity of autism, which can have a significant impact on parenting and the family well-being.

Another related variable was parents’ mental health and family well-being. Parental depression has been linked to behavioral problems in children and decreased quality of family relationships (Xu et al. [44]). The study by Van Steijn et al. [38] related parenting styles to the pathologies of both parents and children. We conclude that children with ASD or ADHD benefited most when their parents shared similar symptoms of these disorders. Furthermore, parents who rate themselves high in depression tend to rate their children’s behaviors as more severe (Clauser et al. [20]). However, studies do not provide solid evidence on how parents’ pathology affects empathy and closeness in the relationship with their children (De Clercq et al. [39]; Hickey et al. [6] and Van Steijn et al. [38]).

Age can influence parenting styles, with a significant increase in stress observed for parents of school-aged children with ASD (Likhitweerawong et al. [29]). Parents encountered more educational challenges at adolescent ages, which was related to a more permissive parenting style and behavioral problems in their children, who benefited from a structured environment (Tripathi, [33]). At an early age, parents used a parenting pattern that oscillated between the role of “coach” and “caregiver”, but as their children grew older, they prioritized “relationships” more (Zhou and Yi [17]). Despite the fact that ASD is more common in boys than in girls, no differences were found in relation to the gender. However, differences were observed in the parenting styles of fathers and mothers. Having high ADHD in fathers and high ASD in mothers was associated with a predominance of the permissive style in relation to the unaffected child (Van Steijn et al. [38]). In general, fathers were less affectionate and warm with their children than mothers (Giannotti et al. [41]). It was also observed that many mothers had a more permissive style than their male partners, and within the group of mothers, those who had children with ASD scored higher on positive parenting compared to mothers of children without ASD (Tripathi [33]).

Culture seems to play an important role. Thus, Japanese fathers present higher levels of stress related to parental competence than Italian fathers, who were more receptive and affectionate. Equally, parents who support a collectivist approach might be more likely to minimize the expression of unpleasant effects or inappropriate childhood initiatives, being perceived as competent by the community (Giannotti et al. [41]). In Indonesia, parents of children with ASD tend to use reasoning less compared to typically developing parents, possibly due to the verbal comprehension difficulties associated with ASD (Riany et al. [37]). Cultural beliefs, such as the idea that having a child with ASD is a punishment for past actions, can limit the support parents of these children receive (Riany et al. [37]). The study by Likhitweerawong et al. [29] suggests that, although there are differences between Thai and Western cultures, the finding of less authoritative and more permissive parenting in children with ASD is similar in both cultures. This may be related to the lack of social relationships due to children’s disruptive behaviors and the stigmatization of having an autistic child, especially in Asian culture.

In summary, family well-being in families with children with ASD is positively associated with authoritarian parenting styles characterized by warmth, relationship building, and low emotional expressiveness (EE). In contrast, authoritarian and permissive parenting styles, as well as behaviors involving harshness and psychological control, are related to higher levels of externalizing behaviors and greater parental stress. High parental stress, especially in mothers, negatively impacts family dynamics, reducing parental involvement and warmth. An authoritative and conscientious parenting style is related to less stress, less perceived discrimination, and fewer behavioral problems in children. Parenting styles are significantly influenced by children’s behaviors and the stress these behaviors generate, often leading to more protective or permissive approaches. The severity of ASD and adolescence are associated with more permissive and authoritarian parenting styles, which contribute to behavioral problems in children. Parental mental health, particularly depression, exacerbates behavioral problems and degrades family relationships. Cultural differences also play a role, with variations in parenting styles and stress levels observed in different cultures. In general, authoritarian parenting leads to better outcomes for both parents and children, while permissive and authoritarian styles are linked to greater stress and behavioral problems.

## 4. Discussion

Our initial objective was to analyze the state of the question of the relationship between the well-being of families with children with ASD and its relationship with parenting styles. We found few studies that considered these three variables (ASD, parenting styles, and family satisfaction or well-being). However, in the final 16 selected articles, it was observed that quality of life and parent–child satisfaction are positively related to authoritative style, warmth, relationship building, affective bonding, priority in relationship building, and low EE.

The comorbidity of ASD and ADHD of parents and children and gender differences are taken into account (Van Steijn et al. [38]). High EE in parents is associated with increased ASD symptoms, which creates a negative cycle (De Clercq et al. [39]) and Cheung et al. [36]). Because the stress-resilient attributes of parents with children with developmental disorders are considered one of the factors that decisively influence the behavior of parents related to raising their children and that influence greater well-being and life satisfaction [9], other factors should be considered to reduce these stress levels, such as children’s internalizing and externalizing symptoms in a bidirectional relationship [20].

The results indicate the need for greater social support, avoiding social isolation, positive feedback on their work as parents, including the rest of the family in the evaluation of well-being, and family respite. Cheung et al. [36] suggest a link between well-being and prosocial behaviors, such that parents who reported greater well-being were more likely to engage in prosocial behaviors. At the same time, children whose parents demonstrated more prosocial behaviors showed more prosocial behaviors [45], and these could serve as a mediating variable for parents’ mental well-being. Single parents had even more difficulties in their social relationships [46]. Likewise, couples who did not recognize the partner’s good practices had more problems as a couple, and their children showed more behavioral problems [42].

It is also important to direct our attention towards prenatal pedagogical education, bearing in mind that the mother influences the education and care of her child before birth [21]. Positive parenting promotes a new attitude by highlighting the importance of educating from affection, having a positive perspective of their parental role, and recognizing that children need a structured, stimulating, and empowering environment away from the use of violence to fully develop [47]. This approach identifies parents’ support needs for positive parenting and works with families to strengthen their capacities to achieve appropriate developmental goals.

The limitations of our review focus on the few studies found with a common criterion in the diagnosis of ASD following the diagnostic criteria of the DSM 5 that included the three main concepts (ASD, parental satisfaction, and parenting style). Our interest was to include articles from the last ten years, coinciding with the conceptual diagnostic change of ASD of the DSM 5; however, some articles, despite being published after this diagnostic update (2013), continued to use previous criteria. Another difficulty was the definition of “parenting style” and the variability of this concept in the existing literature, as well as the concept of satisfaction with the parental role, which was expanded with family well-being and quality of life, although there may be conceptual differences between them. On the other hand, an interjudge agreement coefficient was not calculated that would have served greater rigor in avoiding selection biases. Furthermore, only English language articles were included in this review, likely excluding several studies from non-En glish-speaking countries/published in a different language, which may explain or at least contribute to the lack of diversity in participant ethnicity/race in reviewed studies. Longitudinal studies that take into account demographic factors such as age, socioeconomic level, ethnic origin, and multiculturalism are necessary as they can be of great value for current research on ASD. In addition, it would be interesting to investigate what mechanisms other countries have to address these problems associated with raising children with ASD.

Finally, our search did not include terms such as parenting stress, which may have led to an under-identification of additional articles on the moderating and mediating effects of parenting stress on the association between parenting satisfaction and parenting styles (taking into account the wealth of research on high levels of parenting stress in autistic parents), which can be an important explicit focus in future reviews. On the other hand, it would be advisable to take into account the mediating variables in future studies, such as socioeconomic status, family or psychosocial support, the number of children, and the educational level of the parents, and develop studies that do not use self-reports but rather incorporate other evaluation instruments (observation, third party reports, diaries), for example.

## 5. Conclusions

In conclusion, adopting an authoritarian parenting style with excessive use of punishment and little affection can increase behavioral problems. It seems that the adjustment of parents’ emotional expression, expectations, perceptions, and frustrations is related to a better perception of their children, and they will be more tolerant to their symptoms, which in turn favors the development of their children, the improvement of their symptoms, and the affections in the parent–child relationship. Adopting an authoritative parenting style based on warmth, relationships with children, and conscious and compassionate parenting is associated with lower levels of family stress and greater family well-being.

In view of the results, it seems more than advisable to take care of the mental health and well-being of parents of children with ASD for the benefit of family well-being and even the reduction in symptoms. On the other hand, the most effective parenting style for children with ASD is the same as the parenting style found to be most effective with the wider population of children. This is a finding that will be helpful to clinical practice.

In short, it is necessary to guide the development of specific intervention programs on how to best address the parenting of children with ASD and provide guidance on basic skills and coping strategies. Raising awareness and training parents in more effective and healthy parenting styles could be a line of future work, taking into account the complexity of parenting and the mediators involved in it at different times in the lives of both parents and children.

## Figures and Tables

**Table 1:** Main characteristics of the selected studies.

Author	Participants	Purpose	Results
Walsh et al. [31]	148 mothers of autistic children (2–18 years old)	To evaluate pain and problematic behavior of autistic children as a predictor of parenting behavior and parenting style and stress interaction with pain and behavior.	Greater pain in children increases problematic behavior and It implies more stress in parents and a more overprotective style. If pain is combined with overprotective style, parental stress is higher.
Van Steijn et al. [38]	96 fathers and 96 mothers with possible ASD and/or ADHD; 96 children with ASD+ADHD (2–20 years), siblings not diagnosed with a disability.	To measure the effect of the diagnosis of ASD or ASD+ADHD on undiagnosed children in parenting styles.	More permissive parents with children with ASD. ADHD in fathers and ASD in mothers leads to more permissiveness with non-autistic children.
Xu et al. [44]	33 parents; 24 boys and 9 girls ASD (2.5–5 years old).	To examine the correlation between parental depression and child behavior problems and parenting style mediation in families with children with ASD.	Authoritative style correlated with more behavioral problems and depression than authoritarian and permissive style. Authoritarian and permissive styles alternate, but they do not correlate with behavioral problems in children or depression in parents.
Zhou and Yi [17]	32 fathers and mothers; 27 boys and 1 girl ASD (6.75 mean years)	Understand parenting styles of parents with ASD children and discuss parenting experiences on how to manage symptoms.	Relationship-based style are warmer, less stressed, more emotionally regulated, and more tolerant of the symptoms of children with ASD, as opposed to training-based style, alternation, or self-isolation.
Conti [43]	74 mothers of autistic children and 46 mothers of non-autistic children (5–18 years)	Role of compassionate parenting goals and self-image in the experience of mothers of children with ASD.	Compassionate parenting is more beneficial than self-image-based parenting. Mothers of autistic children showed greater compassionate parenting but lower life satisfaction than mothers of non-autistic children.
Tripathi [33]	320 parents and 320 children with ASD (5–22 years old).	Analyze parenting style of parents with different levels of stress and children with ASD	Mothers are more permissive than fathers. Mothers and fathers are more authoritarian the greater the severity of symptoms. More permissive style in pre-adolescence and higher stress in adolescence.
Riany et al. [37]	388 mothers, 71 fathers and 247 sons and 212 daughters non-autistic children; 86 mothers, 14 fathers and 71 mother of autistic sons and 30 autistic daughters (3–10 years).	To compare parenting styles, parent–child relationships, and social support in parents of autistic and non-autistic children.	Parents of autistic children are more authoritarian, less warm, less relational parenting, and more assertive power in front of a group of non-autistic children. Parents of autistic children have less social support than the group non-autistic children.
Dieleman et al. [30]	15 mothers, 5 fathers, 11 boys and 7 girls with ASD (6–17 years).	Interaction between parenting styles and experiences of parents and how ASD affects the child.	Mutual influence: parenting style (based on autonomy, structure and support in relationships) and feedback from children. The style depends on the needs of the parents.
Cheung et al. [36]	111 mothers, 25 fathers and 111 sons and 25 daughters with ASD (mean 9.39 years).	Role of stress in parental characteristics (disposition to conscious parenting, stigma and mental well-being) and behavioral adjustment of children with ASD.	Less stress promotes mindful parenting, higher quality relationships, greater well-being, fewer behavioral difficulties, and more prosocial behaviors in children with ASD.
Antonopoulou et al. [35]	34 mothers and 16 fathers with ASD; 40 mothers and 10 fathers without ASD (4–12 years).	Correlation between anxiety, expression of emotions, coping strategies, and parenting styles of parents of children with and without ASD.	Parents with ASD had greater negative emotional expression, higher levels of anxiety, permissive style and less authoritative than parents of children without ASD Positive emotional expression predicts coping strategies and supportive parenting styles in both groups of parents.
Hickey et al. [6]	166 stable couples with children with ASD (5–12 years).	To assess whether parental stress and depressive symptoms in parents predict the emotional quality of the relationship between parents and children with ASD	Mothers had more stress and depression than fathers, who predicted more criticism. Greater stress in mothers influences the father’s self-perception of warmth. Increased stress in parents is related to criticism.
Portes et al. [42]	45 fathers and 45 mothers, 39 sons and 44 daughters with ASD (3–7 years).	Correlation between behavior of children with ASD, parenting styles and co-parenting, as a function of the behavior of children with ASD.	Parents of children with behavioral problems present more authoritarian/permissive parenting and negative impacts on co-parenting. Parents of more prosocial children have more authoritative parenting and better quality of co-parenting. Couples who do not recognize good practices in their partners, more relationship and behavior problems in children.
Clauser et al. [20]	66 mothers and 4 father; 70 children (3–18 years) (47% with ASD, 25.7% with Asperger’s Disorder, 27.1% with Pervasive Developmental Disorder (DSM IV-TR), with an average age of diagnosis of 4.84 years	Relationships among parenting style, parenting stress, and behavioral outcomes in children with ASD.	ASD severity and parenting style contributed to externalizing behaviors of children but did not have a significant impact on internalizing behaviors. A supportive an effective parenting style can increase quality parent–child interactions, which in turn is associated with language gains in children with ASD.
Charman et al. [32]	57 mothers, 3 fathers and 2 grandmothers with children with ASD (4–8.11 years).	Feasibility and efficacy of parental behavioral intervention for emotional and behavioral problems in children with ASD.	Lax and hyperreactive practices are associated with increased stress and lower parental well-being.
Giannotti et al. [41]	Group Italy: 47 mothers, 45 fathers, 40 boys and 7 girls ASD. Japan Group: 47 mothers, 42 fathers, 38 boys and 9 girls with ASD (mean 8.9 years).	Differences in parental stress and parenting style between Italian and Japanese mothers and fathers of children with ASD; and the predictive role of culture, sociodemographic, and child characteristics on parental stress and predictors of parenting style.	Japanese fathers (not mothers) have more stress and less commitment in parenting style than Italians. In both cultures: mothers have more social interaction with their children than fathers, and more severe ASD and more stress in parents. Japanese culture, male gender, and stress are related to dysfunctional interaction and predict parenting style.
De Clercq et al. [39]	447 parents: 67 children with Cerebral Palsy (CP) (mean 12.4 years), 54 children with Down syndrome (DS) (mean 13.12 years), 159 children with ASD (mean 10.8 years) and 167 children without disabilities (mean 13.3 years).	To examine the family’s emotional climate and relationship with stress and parental behaviors in ASD, CP, SD and without disabilities.	Parents without disabilities, less Emotion Expressed. Parents of children with disabilities, more responsive parenting. Parents with ASD have more Expressed Emotion, more stress, and hyper-reactive parenting than DS and CP (more critical and less warmth). With and without disabilities, parental warmth is related to children’s well-being.
Likhitweerawong et al. [29]	61 caregivers of children with ASD and 63 without ASD. 49 boys and 12 girls with ASD, 43 boys and 20 girls without ASD (6–12 years).	To assess parenting style, stress, and quality of life of caregivers of children with and without ASD.	ASD caregivers: more stress, depression, anxiety, and lower quality of life; parenting style that is more permissive and authoritarian, and less authoritative than without ASD. Negative correlation between quality of life of children with ASD and authoritarian and permissive parenting styles. More stress with school-age ASD children.
Suvarna et al. [8]	2480 males, 770 females, and two other genders, and 17 no answer. Age range: 2.5 to 24 years old with autistic diagnosis	Associations between parenting practices and externalizing behaviors in autistic children, along with the mediating and moderating effects of parent and child variables	Mindful parenting was associated with fewer or lower levels of externalizing behaviors and negative parenting practices were associated with higher levels of externalizing behaviors.

## Data Availability

The original contributions presented in the study are included in the article, and further inquiries can be directed to the corresponding authors.

## References

[1] Alcantud Marín F., Alonso Esteban Y., Mata Iturralde S. (2016). Prevalencia de los trastornos del espectro autista: Revisión de datos. Siglo Cero.

[2] Abidin R. (1995). Parenting Stress Index.

[3] Dabrowska A., Pisula E. (2010). Parenting stress and coping styles in mothers and fathers of pre-school children with autism and Down syndrome. J. Intellect. Disabil. Res..

[4] Hayes S., Watson S. (2013). The impact of parenting stress: A meta-analysis of studies comparing the experience of parenting stress in parents of children with and without autism spectrum disorder. J. Autism Dev. Disord..

[5] Huang C., Yen H., Tseng M., Tung L., Chen Y. (2014). Impacts of autistic behaviors, emotional and behavioral problems on parenting stress in caregivers of children with autism. J. Autism Dev. Disord..

[6] Hickey E., Hartley S., Papp L. (2020). Psychological well-being and parent-child relationship quality in relation to child autism: An actor-partner modeling approach. Fam. Process.

[7] Greenlee J.L., Piro-Gambetti B., Putney J., Papp L.M., Hartley S.L. (2022). Marital satisfaction, parenting styles, and child outcomes in families of autistic children. Fam. Process.

[8] Suvarna V., Farrell L., Adams D. (2024). Parenting Practices and Externalizing Behaviors in Autistic Children: A System-atic Literature Review. Clin. Child. Fam. Psychol. Rev..

[9] Gavín-Chocano Ó., García-Martínez I., Torres-Luque V., Checa-Domene L. (2024). Resilient Moderating Effect between Stress and Life Satisfaction of Mothers and Fathers with Children with Developmental Disorders Who Present Temporary or Permanent Needs. Eur. J. Investig. Health Psychol. Educ..

[10] Karst J.S., Van Hecke A. (2012). Parent and family impact of autism spectrum disorders: A review and proposed model for intervention evaluation. Clin. Child Fam. Psychol. Rev..

[11] Hock R.M., Timm T., Ramisch J. (2012). Parenting children with autism spectrum disorders: A crucible for couple relationships. Child. Fam. Soc. Work.

[12] Gau S.S., Chou M., Chiang H., Lee J., Wong C., Chou W., Wu Y. (2012). Parental adjustment, marital relationship, and family function in families of children with autism. Res. Autism Spectr. Disord..

[13] Gómez-Marí I., Tárraga-Mínguez R., Pastor-Cerezuela G. (2022). Analysis of Spanish Parents’ Knowledge about ASD and Their Attitudes towards Inclusive Education. Eur. J. Investig. Health Psychol. Educ..

[14] Arellano A., Denne L., Hastings R., Hug J. (2019). Parenting sense of competence in mothers of children with autism: Associations with parental expectations and levels of family support needs. J. Intellect. Dev. Disabil..

[15] May C., Fletcher R., Dempsey I., Newman L. (2015). Modeling relations among coparenting quality, autism-specific parenting self-efficacy, and parenting stress in mothers and fathers of children with ASD. Parenting.

[16] Ventola P., Lei J., Paisley C., Lebowitz E., Silverman W. (2017). Parenting a child with ASD: Comparison of parenting style between ASD, anxiety, and typical development. J. Autism Dev. Disord..

[17] Zhou T., Yi C. (2014). Parenting styles and parents’ perspectives on how their own emotions affect the functioning of children with autism spectrum disorders. Fam. Process.

[18] Shirley M. (1993). The First Two Years. Personality Manifestations.

[19] Sameroff A. (2009). The Transactional Model of Development: How Children and Contexts Shape Each Other.

[20] Clauser P., Ding Y., Chen E.C., Cho S.J., Wang C., Hwang J. (2021). Parenting styles, parenting stress, and behavioral outcomes in children with autism. Sch. Psychol. Int..

[21] Verny T., Kelly J. (1988). La Vida Secreta del Niño Antes de Nacer.

[22] Hernandez-González O., González-Fernández D., Spencer-Contreras R., Tárraga-Mínguez R., Ponce-Carrasco V. (2023). Trends in Autism Spectrum-Related Motherhood Research: A Bibliometric Study. Eur. J. Investig. Health Psychol. Educ..

[23] Costescu C., Roșan A., David C., Cozma L., Calota A. (2023). The Relation between Cognitive and Emotional Processes in Children and Adolescents with Neurodevelopmental Disorders—A Meta-Analysis. Eur. J. Investig. Health Psychol. Educ..

[24] Silver W., Rapin I. (2012). Neurobiological basis of autism. Pediatr. Clin. N. Am..

[25] Baumrind D. (1996). The discipline controversy revisited. Fam. Relat..

[26] Dyches T., Smith T., Korth B., Roper S., Mandleco B. (2012). Positive parenting of children with developmental disabilities: A meta-analysis. Res. Dev. Disabil..

[27] American Psychiatric Association (2013). Diagnostic and Statistical Manual of Mental Disorders: DSM-5.

[28] Page M., Moher D., Bossuyt P., Boutron I., Hoffmann T., Mulrow C. (2021). PRISMA 2020 explanation and elaboration: Updated guidance and exemplars for reporting systematic reviews. Submitted. BMJ.

[29] Likhitweerawong N., Boonchooduang N., Louthrenoo O. (2022). Parenting Styles, Parental Stress, and Quality of Life among Caregivers of Thai Children with Autism. Int. J. Disabil. Dev. Educ..

[30] Dieleman L., Moyson T., De Pauw S., Prinzie P., Soenens B. (2018). Parents’ Need-related Experiences and Behaviors When Raising a Child with Autism Spectrum Disorder. J. Pediatr. Nurs..

[31] Walsh C., Mulder E., Tudor M. (2013). Predictors of parent stress in a sample of children with ASD: Pain, problem behavior, and parental coping. Res. Autism Spectr. Disord..

[32] Charman T., Palmer M., Stringer D., Hallett V., Mueller J., Romeo R., Simonoff E. (2021). A novel group parenting intervention for emotional and behavioral difficulties in young autistic children: Autism spectrum treatment and resilience (ASTAR): A randomized controlled trial. J. Am. Acad. Child Adolesc. Psychiatry.

[33] Tripathi N. (2016). Parenting Style and Parents’ Level of Stress having Children with Autistic Spectrum Disorder (CWASD): A Study based on Northern India. Neuropsychiatry.

[34] González de Dios J., Buñuel Álvarez J., González Rodríguez P. (2012). Listas guía de comprobación de estudios observacionales: Declaración STROBE. Evid. Pediatría.

[35] Antonopoulou K., Manta N., Maridaki-Kassotaki K., Kouvava S., Stampoltzis A. (2020). Parenting and coping strategies among parents of children with and without autism: The role of anxiety and emotional expressiveness in the family. Austin J. Autism Relat. Disabil..

[36] Cheung R., Leung S., Mak W. (2019). Role of Mindful Parenting, Affiliate Stigma, and Parents’ Well-being in the Behavioral Adjustment of Children with Autism Spectrum Disorder: Testing Parenting Stress as a Mediator. Mindfulness.

[37] Riany Y., Cuskelly M., Meredith P. (2017). Parenting Style and Parent–Child Relationship: A Comparative Study of Indonesian Parents of Children with and without Autism Spectrum Disorder (ASD). J. Child. Fam. Stud..

[38] Van Steijn D., Oerlemans A., Ruiter S., van Aken M., Buitelaar J., Rommelse N. (2013). Are parental autism spectrum disorder and/or attention-deficit/Hyperactivity disorder symptoms related to parenting styles in families with ASD (+ADHD) affected children?. Eur. Child Adolesc. Psychiatry.

[39] De Clercq L., Prinzie P., Warreyn P., Soenen B., Dieleman L., De Pauw S. (2022). Expressed emotion in families of children with and without autism spectrum disorder, cerebral palsy and down syndrome: Relations with parenting stress and parenting behaviors. J. Autism Dev. Disord..

[40] Robertson A.E., Simmons D.R. (2015). The sensory experiences of adults with autism spectrum disorder: A qualitative analysis. Perception.

[41] Giannotti M., Bonatti S., Tanaka S., Kojima H., De Falco S. (2021). Parenting Stress and Social Style in Mothers and Fathers of Children with Autism Spectrum Disorder: A Cross-Cultural Investigation in Italy and Japan. Brain Sci..

[42] Portes J.M.R., Vieira M.L., Souza C.D., Kaszubowski E. (2020). Parental styles and coparenting in families with children with autism: Cluster analysis of children’s behavior. Estud. Psicol..

[43] Conti R. (2015). Compassionate Parenting as a Key to Satisfaction, Efficacy and Meaning among Mothers of Children with Autism. J. Autism Dev. Disord..

[44] Xu Y., Cameron L., Parker N. (2014). Parental Depression and Child Behavior Problems: A Pilot Study Examining Pathways of Influence. J. Ment. Health Res. Intellect. Disabil..

[45] Bandura A., Walters R.H. (1977). Social Learning Theory.

[46] Ilias K., Cornish K., Kummar A.S., Park M.S.A., Golden K.J. (2018). Parenting stress and resilience in parents of children with autism spectrum disorder (ASD) in Southeast Asia: A systematic review. Front. Psychol..

[47] Rodrigo López M., Máiquez Chaves M., MartÍn Quintana J., Rodríguez Ruiz B. (2015). Manual Práctico de Parentalidad Positiva.

